# Centromere structure and function: lessons from *Drosophila*

**DOI:** 10.1093/genetics/iyad170

**Published:** 2023-11-01

**Authors:** Eftychia Kyriacou, Patrick Heun

**Affiliations:** Swiss Institute for Experimental Cancer Research (ISREC), School of Life Sciences, Ecole Polytechnique Fédérale de Lausanne (EPFL), 1015 Lausanne, Switzerland; Wellcome Centre of Cell Biology, School of Biological Sciences, University of Edinburgh, EH9 3BF Edinburgh, UK; Department of Biology, Technische Universität Darmstadt, 64287 Darmstadt, Germany

**Keywords:** centromeres, fruit fly, *Drosophila*, chromosomes, epigenetics, FlyBook

## Abstract

The fruit fly *Drosophila melanogaster* serves as a powerful model organism for advancing our understanding of biological processes, not just by studying its similarities with other organisms including ourselves but also by investigating its differences to unravel the underlying strategies that evolved to achieve a common goal. This is particularly true for centromeres, specialized genomic regions present on all eukaryotic chromosomes that function as the platform for the assembly of kinetochores. These multiprotein structures play an essential role during cell division by connecting chromosomes to spindle microtubules in mitosis and meiosis to mediate accurate chromosome segregation. Here, we will take a historical perspective on the study of fly centromeres, aiming to highlight not only the important similarities but also the differences identified that contributed to advancing centromere biology. We will discuss the current knowledge on the sequence and chromatin organization of fly centromeres together with advances for identification of centromeric proteins. Then, we will describe both the factors and processes involved in centromere organization and how they work together to provide an epigenetic identity to the centromeric locus. Lastly, we will take an evolutionary point of view of centromeres and briefly discuss current views on centromere drive.

## Introduction

Model organisms are used to study a particular biological question by taking advantage of a simpler and more tractable system. Nonetheless, in addition to learning about the similarities between species used as model organisms, important insights can be gained by investigating their differences. In this chapter, we are focusing on fly centromeres as a good example for a simple centromere that encompasses both of the above: employing a set of fly-specific components yet serving to ensure accurate chromosome segregation during cell division, a function universally shared among all eukaryotes.

One of the most fundamental processes during the cell cycle is the distribution of equal numbers of the replicated chromosomes into the two daughter cells, to ensure maintenance of the genetic material. Centromeres, loci with specialized chromatin, play a key role in this process. They serve as the platform for the assembly of the kinetochore, a complex proteinaceous structure that is required for centromeric chromatin to attach to the spindle apparatus. Errors in the process of chromosome segregation can lead to aneuploidy, a major cause of miscarriage and a hallmark of cancer. Therefore, to fully elucidate centromere function, it is imperative to understand in great detail their structure and organization.

*Drosophila* has proven to be a powerful model for centromere biology. Fly centromeres are less complex compared to their human counterparts. They contain a much smaller number of constitutively associated centromere proteins, yet they perform the same critical function in chromosome segregation. Importantly, several notions and processes discovered for *Drosophila* centromeres were proven to be simplifications of what holds true in human centromeres and were useful to direct studies carried out in human cells, as we will discuss later.

Here, we are reviewing older and more recent work of various groups that used *Drosophil*a as a model organism and focused on one common theme: understanding the centromere. We will start by describing *Drosophila* centromeres from the DNA sequence to the chromatin composition, how the centromere is assembled, and which proteins associate with it. We will discuss studies that investigated the epigenetic nature of centromeres as well as the evolutionary history of *Drosophila* centromeres and their components. All through, we will be comparing fly centromeres to other species, especially human, highlighting the significance of employing *Drosophila* as a model organism.

## Centromeric DNA in *Drosophila*

**Fig. 1.**
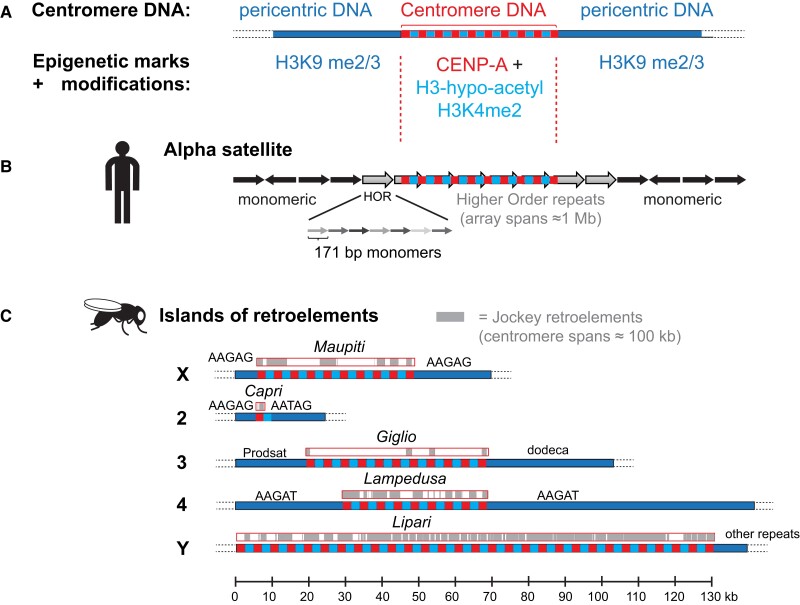
Centromeric DNA in humans and *Drosophila*. a) Domain organization of the centromere DNA and association with epigenetic marks and histone modifications. Note that the functional centromere is defined from the first to the last genomic region occupied by CENP-A (red) and that CENP-A and Histone H3 (light blue) regions are interspersed at the centromere. b) Cartoon of a generic human centromere containing alpha satellite sequences. c) Cartoon of the different fly centromeres containing islands of retroelements interspersed and flanked by simple repeats. Many different types of retroelements are found, but for simplicity, only the Jockey retroelement is shown, which includes the G2/Jockey-3 elements, shared among all centromeres and is the highest enriched sequence for dCENP-A association (Chang *et al*. 2019).

As a functional element of the chromosome, elucidating the DNA composition of centromeres was an essential step to begin the quest to unveiling their function. In the early 70s, it was evident that *Drosophila* centromeric sequences, like in most eukaryotes, are highly repetitive based on hybridization experiments on salivary gland squashes with in vitro transcribed repetitive sequences (Jones and Robertson 1970; Rae 1970; Botchan *et al*. 1971). Parallel studies in mouse and human cells revealed that sequences comprising centromeres in metazoans also contain repetitive sequences (Pardue and Gall 1970; Manuelidis 1978), a centromeric feature that is now accepted to be conserved among many species (Fig. 1). Importantly, the difficulties associated with assembling repetitive DNA into larger sequence reads represented up to recent years a major challenge for the molecular study of centromeres.

### Centromeric sequences obtained by sequencing X-derived minichromosomes

While sequences enriched in human centromeres, namely alpha-satellites, were discovered years ago (Fig. 1b) (Manuelidis 1978) and found to be organized in arrays of higher-order repeats (HORs), studies in *Drosophila* to identify centromeric sequences lagged. However, the use of small, nonessential X-chromosome–derived mini chromosomes that could be fragmented without affecting the fly viability (Karpen and Spradling 1990) allowed for determining the minimal sequences that were necessary and sufficient for inheritance and proper centromere function. Detailed molecular analysis of the X-derived Dp1187 minichromosome centromere suggested that it is primarily composed of the simple repeats AATAT and TTCTC (or AAGAG), interrupted by short islands of complex DNA, named after the Polynesian islands *Maupiti*, *Bora Bora*, *Tahiti*, and *Moorea* and transposable elements (retrotransposons H.M.S. Beagle, 412, BEL, F Doc, and G-like) (Sun *et al*. 1997, 2003; Murphy and Karpen 1995; Le *et al*. 1995). However, these complex X-centromere sequences were not found on all the other autosomal *Drosophila* centromeres (Le *et al*. 1995; Sun *et al*. 1997, 2003), suggesting that unlike in humans, centromeres of different chromosomes are composed of different genetic motifs (Fig. 1c).

### Sequences at native centromeres

Even though minichromosomes provided valuable insight into centromeric DNA sequence structure, the specific composition of centromeric sequences on each chromosome remained elusive. For clarity, in this review, we define the centromere as the underlying DNA sequence spanning from the first to the last genomic region that is associated with the centromere-specific histone H3-variant CENP-A of a single chromosome. The centromere is flanked at either side by large arrays of simple satellite repeats that are organized into pericentric heterochromatin (Fig. 1a). Centromeres of the four different chromosomes in *Drosophila* were studied and compared to understand their evolution and conservation. Mapping of the Y centromere revealed that it has evolved from a telomere, suggesting that an ancestral telocentric chromosome has acquired a pericentric insertion (Méndez-Lago *et al*. 2009). In further support of this, the 18HT satellite present on this chromosome is a tandem array of sequences related to the telomeric retrotransposon-based HeT-A and TART sequences, together with another ancient telomere-related set of complex repeats (Agudo *et al*. 1999; DeBaryshe and Pardue 2011).

Tandem GC-rich arrays called dodeca satellite sequences were shown to be present on the centromere of the third chromosome (Carmena *et al*. 1993; Ferrer *et al*. 1995; Ortiz-Lombardía *et al*. 1998). A 10-mer AATAACATAG satellite called Prodsat is found upstream of the dodeca satellite (Garavís *et al*. 2015) and is bound by the proliferation disrupter protein (Prod) during mitosis, which is thought to play a role in clustering centromeres into chromocenters (Platero *et al*. 1998; Török *et al*. 2000; Jagannathan *et al*. 2019). However, the above studies did not examine specifically which sequences are bound by centromere-specific proteins, like dCENP-A. Thus, using microscopy and native dCENP-A chromatin immunoprecipitation (ChIP), Talbert *et al*. identified sequences specifically bound by dCENP-A, with Prodsat as the most enriched sequence, followed by AATAG, as well as the AATAT sequences. The previously centromere-enriched dodeca satellite and the TTCTC repeats were depleted from dCENP-A precipitates (Talbert *et al*. 2018). Despite this elaborate work, still centromeres were represented as major gaps in genomic assemblies of *Drosophila* chromosomes, not only because of their repetitive nature, like in other organisms, but also because of the lack of information regarding the sequences present on centromeres of different chromosomes.

Major advances in sequencing technologies allowing the production of long sequencing reads enabled the sequencing of all *Drosophila* centromeres in a breakthrough study in 2019 (Chang *et al*. 2019) (Fig. 1c). By assembling long reads of sequences precipitated with dCENP-A, Chang *et al*. found that dCENP-A is assembled over islands of complex DNA sequences, enriched for retrotransposable elements, and flanked by large blocks of simple satellite repeats. The centromeres range between 101 and 171 kb, and while the overall sequence organization of all centromeres is similar, each contains unique islands of complex DNA. Following the nomenclature of Polynesian islands previously exploited for the islands of retrotransposable elements in *Drosophila*, like *Maupiti* for the island present on the X chromosome (Le *et al*. 1995; Sun *et al*. 1997), the newly described islands were named after the Italian islands *Lipari*, *Capri*, *Giglio*, and *Lampedusa* for chromosomes Y, 2, 3, and 4, respectively (Fig. 1c). Interestingly, the non-LTR retroelement *G2/Jockey-3* is a sequence enriched in all centromeres, regardless of its presence in other genomic locations, and associates with dCENP-A. It is noteworthy that even though DNA sequence and organization per se differ between different organisms, it seems that similar to humans, only a portion of the underlying centromeric sequence is occupied by dCENP-A (Sullivan *et al*. 2011; Chang *et al*. 2019).

In addition to its specialized repetitive composition, centromeric DNA was proposed to be fit for engaging in particular conformations. The TTCTC (or AAGAG) satellite can form a parallel pyrimidine triple helix under nearly physiological pH conditions, and this conformation is promoted and stabilized by copper ions (Horn *et al*. 2004; Paris *et al*. 2007). Whether this structure is present in vivo remains to be determined. However, an increase in the number of satellites (as it is the case in vivo) was shown to highly increase the stability of these helices (Horn *et al*. 2004), implying that these structures could be formed and found in vivo (Paris *et al*. 2007). The dodeca satellite can form 4-stranded intercalated structures, called i-motifs, through the C-rich strand. These are composed by 2 parallel duplexes organized in an antiparallel fashion (Garavís *et al*. 2015). Based on the above data and given that centromeric sequences on different chromosomes are not conserved, it is tempting to speculate that the structure of centromeric DNA might contribute to mark a locus for attracting centromeric proteins and be established as the centromere. Advancement of new tools and technologies taking advantage of long-read DNA sequencing will allow for this to be determined in future studies and clarify whether such structures are indeed present in vivo, in endogenous centromeres.

## Centromeric proteins in *Drosophila*

Through the fulfillment of its function as the platform for the assembly of the kinetochore, the centromeric proteome is highly specialized. Starting from the proteins that bind centromeric DNA all the way to the outer kinetochore components that mediate interactions with microtubules, in this section, we will discuss the intriguing complex protein network surrounding *Drosophila* centromeres.

While a centromere-specific H3 histone was identified as marking the centromeres already at the end of the 80s in humans (CENP-A) and budding yeast (Cse4p) (Earnshaw and Rothfield 1985; Palmer *et al*. 1987, 1991; Stoler *et al*. 1995; Meluh *et al*. 1998), its homolog in *Drosophila* (hereafter referred to as dCENP-A) remained unknown for another decade. In 2000, Henikoff *et al*. (2000) described an H3-like protein that localizes specifically to fly centromeres, called Cid, for centromere identifier. Because human and yeast CENP-A have a histone core divergent from canonical H3 as well as a very dissimilar N-terminal tail, they correctly predicted that a *Drosophila* ORF with these characteristics may encode a centromere-specific histone in flies. Indeed, by raising an antibody against the predicted peptide of this ORF, a staining on the primary constriction site of mitotic chromosomes was observed, colocalizing with Polo kinase, a known kinetochore-related protein (Henikoff *et al*. 2000). Subsequent functional studies on dCENP-A confirmed its critical role in centromere and kinetochore function. dCENP-A was shown to be associated with centromeric DNA in or near the inner kinetochore plate and is spatially separated both from outer kinetochore components that are closer to spindle microtubules and pericentric heterochromatin (Blower and Karpen 2001). Interestingly, dCENP-A localization at neocentromeres suggested that its presence correlates with centromere function and activity, independently of the underlying DNA sequence (Blower and Karpen 2001). Indeed, dCENP-A was shown to be required for the recruitment of all outer kinetochore proteins tested and cell-cycle progression.

dCENP-A is profoundly different from canonical H3, and significant identity is only found in the amino acid sequence of the histone fold domain (HFD) (Ahmad and Henikoff 2002). By taking advantage of the adaptive evolution of CENP-A in different *Drosophila* species and the fact that dCENP-A from *Drosophila bipectinata* does not localize to *melanogaster* centromeres, domain swap experiments have identified the loop 1 (L1), within the HFD of dCENP-A, to be a necessary and sufficient element for centromere targeting (Vermaak *et al*. 2002). Several amino acids located at both ends of the L1 loop as well as its minimal length are required for providing centromere targeting (Vermaak *et al*. 2002). As we will discuss later, these evolutionary features of the CENP-A HFD in flies were shown to critically affect the potential deposition of CENP-A into centromeric chromatin, by altering its interaction with its specific loading machinery (Rosin and Mellone 2016).

### The dCENP-A-containing nucleosome

Since centromeric DNA composition did not seem to be the determinant for centromere identity, but rather CENP-A was a common feature between centromeres, the field has seen extensive efforts for the dCENP-A-containing nucleosome structural characterization. Was the presence of a H3 variant in centromeric nucleosomes enough to provide it with specialized properties or was it something else? Studies of the structure of the dCENP-A-containing nucleosomes have yielded contradictory results. Biochemical studies in combination with electron microscopy (EM) and atomic force microscopy (AFM) suggested that dCENP-A nucleosomes have only half the expected height. Based on this data, it was proposed that dCENP-A-containing nucleosomes are heterotypic, containing only one copy of each of the histones dCENP-A, H4, H2A, and H2B, forming a tetramer or “hemisome,” instead of a canonical octameric nucleosome (Dalal *et al*. 2007; Wang *et al*. 2008). Additional experiments led to the proposal that centromeric nucleosomes not only are heterotypic but also wrap DNA right-handedly, inducing positive supercoiling, in contrast to canonical H3-containing nucleosomes (Furuyama and Henikoff 2009). Contrary to these findings, in vivo dCENP-A-containing nucleosomes were shown to contain dCENP-A dimers, suggesting that centromeric nucleosomes are octameric, while this dimerization of dCENP-A was proposed to be essential for correct centromere assembly, further opposing to the suggestion that centromeric nucleosomes were of a “hemisome” nature (Zhang *et al*. 2012). In particular, salt extraction and sucrose gradient ultracentrifugation of centromeric chromatin suggested that centromeric nucleosomes contain two dCENP-A molecules in vivo, consistent with an octameric structure. Importantly, a conserved residue within the 4-helix bundle of dCENP-A was found to play an important role for this dimerization and promoting integrity of dCENP-A-containing nucleosomes (Zhang *et al*. 2012). Copurification of endogenous and tagged dCENP-A from cells retrieves equal amounts of each, suggesting that more than one copy of dCENP-A is present per nucleosome (Erhardt *et al*. 2008), while extended micrococcal nuclease digestions of dCENP-A nucleosomes assembled in vitro by the dCENP-A-specific chaperone suggested that ∼120 bp of DNA is protected, instead of ∼65 bp expected for a half octamer (Chen *et al*. 2014). Thus, nucleosomes containing CENP-A are likely octameric independent of the species they originate from but with specialized physical properties for height and rigidity that distinguish them from nucleosomes containing canonical H3 (Sekulic *et al*. 2010; Miell *et al*. 2013; Ali-Ahmad and Sekulic 2020).

### Centromeric chromatin organization

The CENP-A-containing nucleosome is in fact the major building block of centromeric chromatin. However, using extended chromatin fibers, it was discovered that centromeric chromatin is not solely composed of dCENP-A nucleosomes—as expected from cytological studies of lower resolution (Ahmad and Henikoff 2001)—but rather of interspersed blocks of dCENP-A and H3 nucleosomes, spanning 200–500 kb, with each block comprising ∼14–50 kb (Blower *et al*. 2002). This organization is likely retained throughout the cell cycle, and even in mitosis, as mitosis-specific phosphorylated H3 is also interspersed with dCENP-A and found to be conserved between humans and flies (Blower *et al*. 2002).

Initial reports have demonstrated that like in mammals, fly centromeric chromatin is also embedded in large arrays of heterochromatin (Bonaccorsi and Lohe 1991; Lohe *et al*. 1993; Murphy and Karpen 1995). But even though it is embedded in heterochromatin, which is characterized by the presence of H3K9 dimethylation, this mark is absent from centromeric H3 nucleosomes (Blower *et al*. 2002; Sullivan and Karpen 2004). The boundaries between centromeric and pericentric heterochromatin seem to be sharp with no visible presence of H3K9 dimethylation in the chromatin regions containing dCENP-A (Sullivan and Karpen 2004; Olszak *et al*. 2011; Blower *et al*. 2002; Kharchenko *et al*. 2011; ModENCODE *et al*. 2011). Chromatin at centromeres was also shown to be generally hypoacetylated (Turner *et al*. 1992; Sullivan and Karpen 2004), a feature reminiscent of heterochromatin. However, centromeric H3 nucleosomes are enriched for H3K4 dimethylation—but not H3K4 trimethylation—a mark known to be associated with “open” but not necessarily active chromatin states (Sullivan and Karpen 2004) (Fig. 1a). Thus, histones at the centromere are decorated by both heterochromatic and euchromatic marks, maintaining a unique pattern referred to as “cen-chromatin,” a property also found to be conserved in human centromeres.

How the centromeric chromatin posttranslational modification profile is established, how the boundaries between the 2 types of chromatin are maintained, and their physiological relevance are not clear and remain important questions to be addressed in the future (Fig. 1).

### Factors associating with the dCENP-A nucleosome

As the basis for the complex kinetochore structure, centromeric chromatin interacts with multiple proteins. Several studies attempted to identify fly-specific centromere-associated factors, to extend studies carried out in human cells (Foltz *et al*. 2006). Genetic screens identified interactors of dCENP-A such as the condensin subunit Cap-G (Jäger *et al*. 2005) or the long sought-after homolog of CENP-C, a constitutive centromere protein, colocalizing with dCENP-A during interphase and mitosis (Heeger *et al*. 2005). dCENP-C's localization to the centromere is mediated by its C-terminal domain, specifically through a region encompassing amino acids 1009–1205 that is sufficient to target the centromere (Heeger *et al*. 2005). Contrary to the fly homolog, mammalian CENP-C has been shown to bind CENP-A nucleosomes through its central domain (amino acids 426–537) (Carroll *et al*. 2010; Kato *et al*. 2013) and its CENP-C motif (727–767) (Kato *et al*. 2013; Ali-Ahmad *et al*. 2019). dCENP-C shows moderate to low sequence conservation with the central domain (19% identity), but its CENP-C motif is highly similar to other species (amino acids 1086–1117, 29% identity with human) suggesting a similar ability to bind CENP-A nucleosomes directly. Despite some differences in its C-terminal domain, dCENP-C is highly conserved between Drosophilids, particularly at its N-terminus (Heeger *et al*. 2005). Interestingly, similar to its budding yeast homolog Mif2 but unlike human, dCENP-C also contains an AT hook. In Mif2, the AT hook is part of the DNA- and histone-binding domain (DHBD) (Xiao *et al*. 2017) and contributes to centromere binding, but whether it performs a similar role in dCENP-C remains to be shown (Heeger *et al*. 2005).

**Fig. 2.**
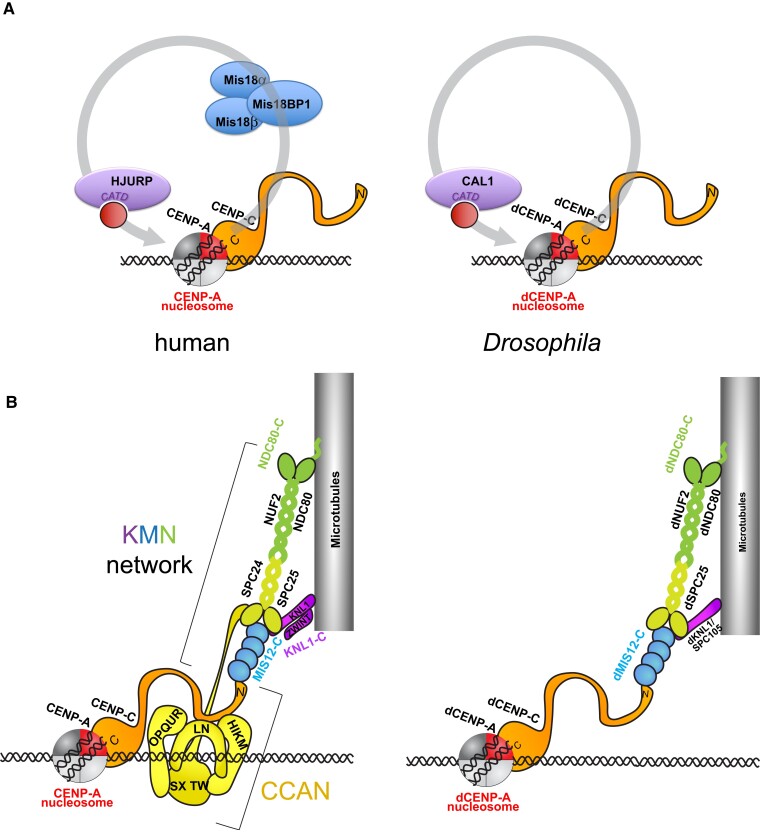
CENP-A maintenance and kinetochore organization in human and *Drosophila*. a) Cartoons show the factors involved in the epigenetic inheritance of CENP-A. Human CENP-C is associated with a CENP-A nucleosome and recruits the MIS18 complex, which in turn targets the CENP-A chaperone holliday junction recognition protein (HJURP) to centromeres to load new CENP-A. dCENP-C directly recruits the dCENP-A chaperone CAL1 to load new dCENP-A to centromeric chromatin. b) Cartoons show the kinetochore organization at the centromere in mitosis with connection to spindle microtubules. CCAN is shown in orange (CENP-C) and yellow (HIKMLNOPQURSXTW); the KNL1-MIS12-NDC80 (KMN) network is shown in purple (KNL1-complex), blue (MIS12-complex), and light and dark green (NDC80-complex). Note that dCENP-C is the only CCAN member in *Drosophila*.

Attempts to identify proteins possibly homologous to the Constitutive Centromere Associated Network (CCAN) (Foltz *et al*. 2006) (Fig. 2b; Table 1) by analyzing the interactome of the immunoprecipitated dCENP-A nucleosome have failed to pinpoint proteins exclusively and constitutively present at centromeres (Barth *et al*. 2015, 2014). Nonetheless, other factors enriched at centromeres were identified, including the histone acetyltransferase 1 (HAT1) homolog, the proteasome regulator REG, and the transcription elongation factor Spt6 (Barth *et al*. 2014; Bobkov *et al*. 2020). Curiously, although the possibility remains that functional homologs of the CCAN proteins are yet to be discovered, it appears that *Drosophila* has evolved a very reduced inner kinetochore, with dCENP-C as the only component fulfilling all functions of the complex and multiprotein CCAN human counterpart (Fig. 2c; Table 1).

**Table 1.** List of *Drosophila* centromere proteins including mention of components missing compared to human centromeres.

	Protein name	Family	Functions at centromeres	References
**Centromeric chromatin**	dCENP-A	Histone H3 variant	Replaces canonical H3 and wraps a portion of centromeric DNA to nucleosomes Specifies centromeric identity	Earnshaw and Rothfield (1985) Henikoff *et al*. (2000)
**CCAN (inner kinetochore)**	dCENP-C		Binds the dCENP-A nucleosome and bridges centromeric chromatin to outer kinetochore proteins Interacts and recruits Cal1 to centromeres for dCENP-A propagation (only CCAN member conserved in *D. melanogaster*)	Heeger *et al*. (2005)
		**Members**		
**KMN network (outer kinetochore proteins)**	MIND/dMIS12 complex	dMIS12, NSL1, NNF1a, NNF1b	Binds dCENP-C, KNL-1 and dSPC25 (DSN1 not found)	Przewloka *et al*. (2007) Schittenhelm *et al*. (2007)
	dNDC80 complex	dSPC25	Interacts with dMIS12 (SPC24 not found)	
		dNDC80	Interacts with dSPC25 and microtubules	
		dNUF2	Interacts with dNDC80 and dSPC25	
	SPC105/KNL-1		Anchors the dMIS12 complex to microtubules (ZWINT not found)	
**CENP-A assembly**	CAL1	dCENP-A chaperone	Incorporates dCENP-A into centromeric nucleosomes (functional homolog of HJURP)	Goshima *et al*. (2007) Erhardt *et al*. (2008)

A breakthrough in our understanding of CCAN association with human centromeric nucleosomes has recently been achieved through CryoEM studies (Fig. 2b; Pesenti *et al*. 2022; Yatskevich *et al*. 2022). Interestingly, the structure of the CCAN bound to the CENP-A nucleosome by the Barford Lab reveals a tight association of CCAN proteins with linker DNA protruding from the CENP-A nucleosome. It is suggested that this type of anchoring might help to stabilize the centromere structure on chromatin, when exposed to the forces exerted by the mitotic spindle. Which proteins might play a similar function in *Drosophila* remains unclear at this point, but dCENP-C would be a potential candidate to fulfill this role.

Unlike the CCAN, outer kinetochores or KNL1-MIS18-NDC80 (KMN) network is more conserved in *Drosophila* (Fig. 2b) (Przewloka *et al*. 2007; Schittenhelm *et al*. 2007; Liu *et al*. 2016). Numerous approaches have been taken to characterize them in molecular detail. Phylogenetic analyses of the eukaryotic kinetochores have identified potential orthologs of the outer kinetochore members Mis12, Ndc80, and Nuf2 (Meraldi *et al*. 2006), which were afterward confirmed to be proteins colocalizing with dCENP-A at centromeres (Przewloka *et al*. 2007; Schittenhelm *et al*. 2007; Fig. 2b). A proteomic-based approach using tagged proteins of known members of the outer kinetochore components Mis12, Ndc80, and Nuf2 proposed that the three proteins often copurify, in addition to identifying novel factors, probably members of the potential *Drosophila* MIND/MIS12, NDC80, and SPC105/KNL-1 complexes (Przewloka *et al*. 2007; Schittenhelm *et al*. 2007). Those are assembled sequentially, with the MIND/Mis12 complex being recruited to the centromeres first by the core kinetochore proteins dCENP-A/CENP-C, followed by the recruitment of the NDC80 complexes. In yeast and human, the microtubule-binding Ndc80 complex contains four proteins (NCD80, NUF2, SPC24, and SPC25) (DeLuca *et al*. 2006; Wei *et al*. 2007; Ciferri *et al*. 2008). A canonical Spc24 homolog could not be identified in *Drosophila* (Cheeseman *et al*. 2006; Przewloka *et al*. 2007; Schittenhelm *et al*. 2007), and only SPC25 appears to anchor the NDC80 complex to the underlying KNL1 SPC105/KLN-1 and MIND/MIS-12 complex (Przewloka *et al*. 2007). dCENP-C is closer to centromeric chromatin and dCENP-A via its C-terminus, which also contains the region responsible for dCENP-A interaction, and its N-terminus extends to the outer kinetochore (Schittenhelm *et al*. 2007; Przewloka *et al*. 2011). Closer to dCENP-C N-terminus is the dSPC25 subunit, followed closely by dMIS12, while in the outermost surface, and possibly closer to the microtubules, are dNUF2 (Schittenhelm *et al*. 2007) and dNDC80 (Fig. 2b; Table 1).

The quantitative architecture of *Drosophila* kinetochores has also been explored. Counting the number of molecules per kinetochore of different kinetochore components (dSPC105, dMIS12, ddSPC25, and NUF2) (Fig. 2b) has suggested that these proteins are present in similar amounts, lower than the amount of dCENP-A at the centromere, and about half the amount of dCENP-C, which can dimerize (Schittenhelm *et al*. 2010). Interestingly, these numbers seem to correlate with the calculated number of kinetochore microtubules in flies, and this correlation is consistent and suggests conservation with observations in yeast kinetochores (Maiato *et al*. 2006; Schittenhelm *et al*. 2010).

The above studies provided insights in the layered structure of the kinetochore and its quantitative organization. *Drosophila* seems to employ a simplified version of an inner kinetochore, but it appears that the outer kinetochore layer and the one directly responsible for mediating attachments to spindle microtubules is largely conserved and resembles the one from other species.

### 3D structure of the kinetochore

Centromeric chromatin constitutes the platform for the assembly of the kinetochore, and despite its 2D organization being elucidated, its 3D organization has not been extensively studied yet. Only a few models have been proposed for the 3D organization of the centromere and the kinetochore. Early reports based on a repeat subunit model (Zinkowski *et al*. 1991) proposed that centromeric chromatin might be arranged in a spiral solenoid or a looping structure, in which dCENP-A-containing blocks are exposed to the poleward faces of the chromosomes, allowing interactions with the CCAN and eventually outer kinetochore proteins, while the H3-containing nucleosome blocks are facing the inner centromere region in the space between the 2 sister chromatids (Blower *et al*. 2002). EM experiments on human centromeres and neocentromeres suggested that the 3D organization of the primary constrictions of chromosomes is perhaps even more complex, possibly implicating further coiling or folding of the coiled centromeric chromatin, to achieve a higher-order compact structure (Marshall *et al*. 2008). The most recent model that was put forward for the 3D organization of centromeres is taking into account all the above, and based on results from chicken DT-40 cells and stretched kinetochore fibers, kinetochores are proposed to be arranged in a “boustrophedon” model (Ribeiro *et al*. 2010). In such a model, centromeric chromatin is anticipated to be arranged in a sinusoidal layered arrangement, linked to heterochromatin on each side, and allowing for interactions with the CCAN and outer kinetochore members in the outer surface (Ribeiro *et al*. 2010). A key feature of all models for the higher-order organization of the centromere is that outer kinetochore components and pericentric heterochromatin bound by HP1 are distinct domains (Blower and Karpen 2001). Interestingly, pericentric heterochromatin appears to be located between two sister kinetochores and in the chromatin immediately flanking the centromere (Sullivan and Karpen 2004).

The localization pattern of specific kinetochore members on mitotic chromosomes was visualized in high resolution to decipher their specific spatial arrangement. Interestingly, dCENP-C does not have the same pattern as dCENP-A as seen on mitotic kinetochores imaged in high resolution. It colocalizes with dCENP-A at the core of the kinetochore and is seen as two discrete foci on two sister chromatids; however, it seems to extend further than the dCENP-A discrete dot, possibly reaching to the outer kinetochore (Blower *et al*. 2002; Maiato *et al*. 2006; Schittenhelm *et al*. 2007). Unlike dCENP-C, outer kinetochore proteins like CENP-E were found to be forming a layer above the dCENP-A spot, and the whole kinetochore forms a bilaminar hemispheric structure (Maiato *et al*. 2006). Although the molecular details regarding the interactions of centromeric chromatin and kinetochore proteins have been extensively demonstrated, it remains an open question how exactly centromeric chromatin is folded in vivo in interphase (since most of the above studies focused on mitosis) and how this organization is maintained before and after cell division. In addition, whether heterochromatin flanking the complex kinetochore structure plays any role to the 3D arrangement or functionality during mitosis remains to be determined.

## Epigenetic identity of centromeres

### dCENP-A is sufficient for centromere identity

While centromeric DNA sequences show little conservation between different organisms, CENP-A is present across many species in the opisthokont (including fungi and animals) and plant group of eukaryotes. It was therefore speculated that CENP-A could play an important role in determining centromere identity. Indeed, it has now been extensively demonstrated that centromeres in most organisms—with the exception of budding yeast—are epigenetically defined (Karpen and Allshire 1997), with CENP-A being the central epigenetic mark.

In support of this, the plasticity and spreading of a centromere mark were particularly highlighted in studies using the propagation of chromosome fragments after X-ray irradiation in *Drosophila*. Neocentromeres only formed on fragments that were located proximally to endogenous centromeres, suggesting that centromere identity can spread to nearby DNA sequences (Maggert and Karpen 2001). Indeed, it was later shown that mislocalization of dCENP-A promotes the formation of functional kinetochores both in cells and animals (Heun *et al*. 2006), contradicting studies performed in human cells where overexpression of CENP-A was not sufficient to induce formation of functional ectopic kinetochores (Hooser *et al*. 2001). This induction of functional ectopic kinetochores led to chromosome segregation defects, resulting in aneuploidy and growth defects (Heun *et al*. 2006). Overexpressed dCENP-A is incorporated in euchromatic regions but not in pericentric heterochromatin, supporting a previous hypothesis that heterochromatin antagonizes the spreading of centromeric chromatin, controlling its size and distribution (Maggert and Karpen 2001; Heun *et al*. 2006). Heterochromatin does, however, promote de novo functional centromere formation, as dCENP-A-overexpression–mediated functional kinetochores preferentially assemble close to pericentric heterochromatin or telomeres (Olszak *et al*. 2011), in agreement with findings in *Schizosaccharomyces pombe* and attempts for building human artificial chromosomes (Barrey and Heun 2017).

Further supporting a central role for CENP-A in conferring centromere identity was the finding that artificial tethering of dCENP-A to an ectopic site is sufficient to promote functional kinetochore formation as well as self-propagation and inheritance of the epigenetic mark (Mendiburo *et al*. 2011), alongside similar findings in human cells and in vitro (Barnhart *et al*. 2011; Guse *et al*. 2011). Using a heterologous system to dissect the molecular determinants for *Drosophila* centromere identity, it was shown that the three factors dCENP-A, dCENP-C, and the chaperone Cal1, are sufficient to promote heritable centromere identity, implying a 3-component epigenetic loop for centromere propagation (Roure *et al*. 2019).

### dCENP-A inheritance

dCENP-A is present at centromeres in postmeiotic spermatids (Dunleavy *et al*. 2012; Raychaudhuri *et al*. 2012), suggesting that it survives the nucleosome replacement by protamines during spermatogenesis. It also serves as a mark for the transgenerational inheritance of centromere identity, as well as being important for the quantity of dCENP-A loaded to the centromeres, as seen in studies where dCENP-A levels were manipulated in males and the levels of dCENP-A on paternal chromosomes were quantified in the offspring (Raychaudhuri *et al*. 2012). Those altered dCENP-A levels were inherited on the paternal chromosomes of the offspring throughout development. When dCENP-A was eliminated in the males, dCENP-A was not present at the centromeres of paternal chromosomes of the embryos. Altogether, these data suggest that already present dCENP-A is a prerequisite for the recruitment of more dCENP-A, but it also regulates the amount of more dCENP-A that is recruited (Raychaudhuri *et al*. 2012).

### Timing of dCENP-A assembly

Unlike canonical H3, dCENP-A incorporation into chromatin is replication independent (Ahmad and Henikoff 2001; Sullivan and Karpen 2001; Blower *et al*. 2002; Schuh *et al*. 2007). Following mitosis, dCENP-A is equally distributed to the daughter cells, as evinced by pulse-chase experiments after one cell division where a close to 50% decrease of labeled dCENP-A levels is measured (Mellone *et al*. 2011; Bobkov *et al*. 2020). The timing of dCENP-A assembly into centromeres was first studied by live imaging of syncytial embryos, where it was suggested that dCENP-A is incorporated into centromeric chromatin during anaphase (Schuh *et al*. 2007). The assembly of dCENP-A during that time is accompanied by an increase in dCENP-C levels at the centromeres, and the whole process requires progression through mitosis (Schuh *et al*. 2007). In Kc cells, dCENP-A assembly was shown to be tightly coupled to the cell cycle, since cyclin A and regulator of cyclin A1 (RCA1) depletion was shown to influence dCENP-A localization to the centromere (Erhardt *et al*. 2008). In human cells, the timing of CENP-A deposition has been determined to start at the end of mitosis in anaphase and continue through G1 (Jansen *et al*. 2007). For *Drosophila*, the exact timing of dCENP-A deposition remains controversial. Studies in *Drosophila* cell culture cells (S2 and Kc) proposed that dCENP-A loading occurs only in mitosis, specifically starting already in metaphase, following the increase of Cal1 at centromeres during prophase (Mellone *et al*. 2011; Pauleau *et al*. 2019). However, in vivo imaging of EGFP-tagged dCENP-A revealed that its loading in S2R+ cells occurs mostly during early G1 with only a transient increase in dCENP-A levels at centromeres during metaphase (Lidsky *et al*. 2013). This is similar to observations in somatic tissues of the fly (Dunleavy *et al*. 2012) and the release of cytoplasm-anchored dCENP-A in S2 cells (Bobkov *et al*. 2018), matching more closely the window for CENP-A loading in human cells (Jansen *et al*. 2007; Dunleavy *et al*. 2012; Lidsky *et al*. 2013; Bobkov *et al*. 2018). Interestingly, in meiosis, dCENP-A assembly timing is different than mitosis of cultured cells and somatic tissues, since, in females, it occurs during prophase I while, in males, it occurs in 2 stages, during prophase I and after exit from meiosis II, in spermatids (Dunleavy *et al*. 2012).

### Proteins involved in dCENP-A assembly and regulation

In humans, CENP-A assembly is mediated by a complex set of proteins priming chromatin before incorporation of CENP-A. This involves the CENP-A-specific chaperone holliday junction recognition protein (HJURP) and CENP-C and connecting these two proteins, the Mis18 complex, which is composed of Mis18α, Mis18β and Mis18BP1 (McKinley and Cheesman 2014)(Fig. 2a). *Drosophila* instead seems to have simplified this process for achieving the same goal. To identify the factors mediating dCENP-A assembly into centromeric chromatin, different approaches have been taken in multiple studies. First, the complex interacting with soluble dCENP-A in S2 cells was described to contain H4 and the chaperone RbAp48, and the latter was shown to be able to assemble dCENP-A-containing nucleosomes in vitro (Furuyama and Henikoff 2006). It was earlier shown that *argonaute-2* mutant embryos have defects in dCENP-A assembly, since they presented lower levels of dCENP-A at centromeres, suggesting that the RNAi machinery might play a role in this process (Deshpande *et al*. 2005). In a genome-wide RNAi screen looking for factors affecting dCENP-A centromeric localization, dCENP-C and Cal1 were the two proteins showing the strongest effect, along with the cell-cycle regulators cyclin A and the RCA1 (Erhardt *et al*. 2008). A previous study had already identified Cal1 as a factor important for centromeric localization of dCENP-A and dCENP-C (Goshima *et al*. 2007), and depletion of dCENP-C also impaired dCENP-A localization (Orr and Sunkel 2011).

As studied by in vitro nucleosome assembly assays, Cal1 can specifically assemble left-handed octameric nucleosomes containing dCENP-A through its N-terminal domain and was therefore called the dCENP-A-specific chaperone (Chen *et al*. 2014). Interestingly, tethering of Cal1 to an ectopic site is also sufficient to promote dCENP-A and kinetochore assembly, similar to what was observed in tethering experiments of HJURP (Hori *et al*. 2013; Chen *et al*. 2014; Palladino *et al*. 2020). Curiously, unlike HJURP, Cal1 is present at centromeres throughout the cell cycle, almost constitutively, in addition to the nucleolus, where it was suggested to be sequestered through its middle region (Erhardt *et al*. 2008), when not in complex with dCENP-A and dCENP-C (Schittenhelm *et al*. 2010; Lidsky *et al*. 2013) (Fig. 2a). The interactions between Cal1, dCENP-A, and dCENP-C were further dissected by yeast 2- and 3-hybrid assays, which showed that Cal1 interacts via its N-terminus with dCENP-A, while its C-terminus interacts with the C-terminus of dCENP-C. Since Cal1 can directly interact with dCENP-A and dCENP-C, it was suggested that it might be a bridging factor between the 3 proteins at centromeres (Schittenhelm *et al*. 2010). To answer the question of whether Cal1 acts as a stoichiometric bridge between dCENP-A and dCENP-C molecules, the number of molecules per centromere was counted in wing imaginal discs. Interestingly, it was found that while dCENP-A is present in 84 copies per centromere, dCENP-C was present as 135 molecules and Cal1 as only 2.5 molecules per centromere, excluding the possibility that all dCENP-A and dCENP-C molecules are bridged by Cal1 (Schittenhelm *et al*. 2010).

Together, the N- and C-terminal regions of Cal1, responsible for interaction with dCENP-A and dCENP-C respectively, are required for its centromeric function (Schittenhelm *et al*. 2010), and its N-terminus was shown to contain an “Scm3 domain”-like region (Phansalkar *et al*. 2012), suggesting possible conservation in the domain organization of Cal1, as in CENP-A chaperones in other species. Despite its evolutionary uniqueness, Cal1 participates in a process that is evolutionarily conserved, that is, however, as it seems, highly plastic. Even though Cal1 is structurally distinct from other CENP-A chaperones, and especially the human ortholog HJURP, a recent study reporting on the crystal structure of Cal1 together with dCENP-A-H4 and dCENP-C surprisingly suggests that Cal1 engages with its substrates using modes employed by both HJURP and Smc3 (Cho and Harrison 2011; Hu *et al*. 2011; Chik *et al*. 2019; Medina-Pritchard *et al*. 2020).

In addition to Cal1, soluble dCENP-A was found to interact with Modulo (Chen *et al*. 2012), the chaperones Caf1 and FACT, and Hat1, which binds it in a complex distinct from the Cal1/FACT-CENP-A-H4 complex, but together with acetylated H4 and Caf1 (Boltengagen *et al*. 2015). This interaction between dCENP-A and Hat1 does not affect its acetylation status but rather was shown to play a role in dCENP-A assembly.

In summary, it appears that Cal1 or dCENP-C in *Drosophila* fulfills some functions of the Mis18 complex in human cells, yet the molecular details of their action remain to be established.

### Regulation of dCENP-A assembly

A crucial question in the field has been how the deposition of the histone H3 variant CENP-A is restricted specifically to the centromeres and how it is replenished after DNA replication. While dCENP-A is expressed in the early S phase (Henikoff *et al*. 2000), its levels and availability are effectively regulated by proteasomal degradation, and even overexpressed and mislocalized dCENP-A will be constrained at the centromeres within a few cell cycles (Moreno-Moreno *et al*. 2006; Olszak *et al*. 2011). Indeed, the F-box protein, partner of paired (Ppa), was found to interact with the L1/α2 of the HFD of dCENP-A, in both soluble and nucleosomal states, and regulate its stability (Moreno-Moreno *et al*. 2011). Ppa is a component of the E3 ubiquitin ligase SCF, which interestingly is inactive during mitosis (Nakayama and Nakayama 2006), the preferred cell-cycle stage for dCENP-A incorporation into centromeric chromatin. This suggests that a tight regulation for specific incorporation of dCENP-A only at centromeres is achieved because dCENP-A is only made available in mitosis when its proteolysis is inhibited. A fine balance between dCENP-A degradation and availability during a specific cell-cycle stage ensures that dCENP-A is not misincorporated elsewhere in the genome and that it is restricted only at centromeres. Proteasome-mediated degradation of cyclin A and other targets is required for dCENP-A assembly in *Drosophila* (Mellone *et al*. 2011).

To add another level of regulation in dCENP-A assembly, Cal1 was found to interact directly with RDX, a protein that acts as an adaptor for CUL3-mediated ubiquitinylation. Cal1 is not itself a target of the complex; it rather helps in specifying dCENP-A as a substrate, and interestingly, ubiquitinylation of the latter does not promote its degradation but rather stabilizes it together with Cal1 (Bade *et al*. 2014). CHRAC14, another interacting factor of dCENP-A, prevents its incorporation at sites of DNA damage (Mathew *et al*. 2014).

Like dCENP-A, dCENP-C is also equally distributed to daughter cells while Cal1 is turned over at a higher rate per cell cycle (66% of preexisting Cal1 is replenished in each cell cycle) (Mellone *et al*. 2011). Cal1 is highly dynamic at centromeres, with its levels being decreased during progression to the G1 phase but increase again in the transition between G1 and S phases and do not change during S phase and G2 (Lidsky *et al*. 2013). During the late M phase, early G1, when dCENP-A loading occurs, centromeric Cal1 levels remain high (Lidsky *et al*. 2013). In contrast, dCENP-C is mostly stably incorporated at centromeres with some fluctuation of its levels seen throughout the cell cycle. dCENP-C was found to be increased mainly during the late S phase, G2, and mitosis, differently from dCENP-A and also differently from the timing of its centromere loading in early embryos (Schuh *et al*. 2007; Lidsky *et al*. 2013). While Cal1 dynamics correlate with the time dCENP-A loading occurs, it remains to be determined what is the physiological relevance of the changes in dCENP-C levels throughout the cell cycle. Particularly, does the increase seen during the late S phase, G2, and M play any role in ensuring dCENP-A stability/maintenance of centromeric position after dilution of dCENP-A nucleosomes following DNA replication?

### Centromeres are transcriptionally active, and this affects dCENP-A assembly

Several hypotheses attempted to explain the preferential incorporation of dCENP-A over H3 into centromeric nucleosomes. One such proposal was that centromeres are replicated with different timing compared to other loci, allowing for the distinction of the sites where dCENP-A should be assembled (Ahmad and Henikoff 2001). Although an attractive model, experiments using different pulse labeling and chase timings showed that centromeres are asynchronously replicated in the mid and late S phase in *Drosophila* tissue culture cells and larval neuroblasts, suggesting that replication timing is not the determinant of centromeric nucleosome assembly (Sullivan and Karpen 2001).

dCENP-A is distributed to the two sister centromeres during every S phase, and gaps emerge at sites previously occupied by dCENP-A. These are possibly filled by placeholder H3/H3.3 nucleosomes as it has been suggested by studies in human cells and fission yeast (Dunleavy *et al*. 2011; Shukla *et al*. 2018). Histone deposition during the S phase is easily realized since gaps are being formed that can be subsequently filled by new histones. Since CENP-A deposition in *Drosophila*, and most organisms, is uncoupled from DNA replication, it is hard to envision how dCENP-A can be deposited at centromeres. Recent studies in accordance with the notion that fission yeast and human centromeres are transcribed (Choi *et al*. 2011; 2012; Chan *et al*. 2012; McNulty *et al*. 2017) have shown that similarly fly centromeres are transcribed and that FACT-mediated transcription is important for dCENP-A assembly in somatic cells (Roši *et al*. 2014; Chen *et al*. 2015; Bobkov *et al*. 2018). This provides an attractive model for the specific incorporation of dCENP-A at centromeres. dCENP-A becomes available during mitosis when chromatin is largely compacted. Conveniently, centromeres are specifically transcribed during mitosis, potentially evicting H3 placeholder nucleosomes, allowing for the generation of chromatin gaps for the specific incorporation of dCENP-A nucleosomes. It is important to note that during early *Drosophila* embryogenesis, the loading of dCENP-A does not require RNAPII-mediated transcription (Ghosh and Lehner 2022). It is tempting to speculate that this might reflect more open, nucleosome gap-containing chromatin because of the very fast nuclear divisions in early embryogenesis, while somatic chromatin might have more time for full nucleosome occupancy and hence be more “sealed” for dCENP-A incorporation. Moreover, it is still unclear whether the act of transcription per se or the RNA products are important for dCENP-A incorporation, and this remains to be experimentally determined. However, that transcription-mediated chromatin remodeling can evict nucleosomes at the centromere, including those containing old dCENP-A, has been recently demonstrated in cells depleted for the transcriptional chaperone Spt6 that serves as CENP-A maintenance factor (Bobkov *et al*. 2020).

## *Drosophila* as a model organism to understand neocentromeres and evolution

### Lessons from neocentromeres and dicentric chromosomes

Concurrently, with neocentromere cases being reported in human cells (du Voullaire *et al*. 1993; Depinet *et al*. 1997; Sart *et al*. 1997), a study using minichromosomes lacking centromeric sequences in *Drosophila* aimed at understanding the relationship between DNA sequence and centromere function (Williams *et al*. 1998). Interestingly, these seemingly structurally acentric minichromosomes were recovered after irradiation mutagenesis (Murphy and Karpen 1995), even though they are lacking centromeric sequences, raising the possibility that they acquire centromere functions and thus acquire neocentromeres (Williams *et al*. 1998). Indeed, these minichromosomes were not lost upon cell division, mostly in male meiosis and less in mitosis and female meiosis. They were shown to bind outer kinetochore proteins like ZW10 and the anaphase spindle equally well as endogenous centromeres, and when checked, they were negative on centromeric DNA sequences (Williams *et al*. 1998). Several lines of evidence suggest that there are no specific sequence characteristics that would determine centromere function; however, the specific chromatin state of a region was suggested to play a role in the specification of centromeres. Particularly, Henikoff and colleagues (Platero *et al*. 1999) using flies containing a distal heterochromatic block on the right arm of their second chromosome demonstrated that this heterochromatic block occasionally acquires centromere activity, suggesting that even though sequence per se is not capable of determining centromere identity, heterochromatic blocks composed of satellites display centromere competence (Platero *et al*. 1999). *Drosophila*, in contrast to mammalian systems, not only served as a powerful tool to study both neocentromere formation but also allowed for manipulation of the system.

While natural selection favored either monocentric or holocentric chromosomes, the presence of intermediate variants usually leads to breakage–fusion events, causing major chromosomal rearrangements. Interestingly, a dicentric transmissible chromosome was recovered in *Drosophila* (Y S · Y L 2Rh4), which consists essentially of the entire Y and fourth chromosomes joined by 2R heterochromatin, even though earlier studies have suggested that this is not possible (Ault and Lyttle 1988). It was discussed that this dicentric was recovered due to differences in strength of the two centromeres and that since the Y chromosome might have a stronger kinetochore has contributed to the recovery and not breakage of this dicentric chromosome (Ault and Lyttle 1988). Another dicentric chromosome C(1)A was recovered in flies, with one of the two centromeres carrying all the sequences of the centromere of the Y chromosome and the other carrying only a part of the Y centromeric region, rich in telomere-related sequences (Agudo *et al*. 2000). Similar to what has been reported in other organisms (Fisher *et al*. 1997; Sullivan and Wilard 1998), one of its two centromeres is inactivated in most cases. Interestingly, in the less frequent cases of dicentrics, one of the two kinetochores is more faintly stained than the other, suggesting that it is possible that kinetochore strength might play a role in the successful segregation of dicentric chromosomes (Agudo *et al*. 2000). Importantly, the above studies exhibit strong lines of evidence that indeed centromeres are epigenetically, rather than genetically specified. Intriguingly, the same principles seem to be conserved across species, except for *Saccharomyces cerevisiae*, where there is a specific centromeric sequence determining centromere position.

### *Drosophila* centromeres as a model to understand meiotic drive

The hypothesis for a meiotic drive in *Drosophila* was put forward by Sandler and Novitski back in 1957 (Sandler and Novitski, 1957), as an attempt to explain a phenomenon in which species create certain gametes in unequal frequencies. Such an occurrence is common in female gametes of most animals and plants, where, of the four meiotic products, only one survives and makes it to the oocyte (Henikoff and Malik 2002; Rosin and Mellone 2017). Centromeres were characterized as “selfish” genomic elements, which, by taking advantage of the meiotic drive, try to make it into the oocyte. Rapidly evolving centromeres that usually acquire expanded satellite arrays were proposed to have an advantage over smaller centromeres, since they can attract more kinetochore proteins, resulting in their kinetochores being “stronger” and thus more likely to attach on the spindle microtubules and make it to the oocyte. In the female meiosis, this is advantageous to the “newly expanded” centromere; however, this could have deleterious consequences in male meiosis, since such a discrepancy in the size and sequence of the evolved centromere would not allow for efficient homolog pairing and lead to increased nondisjunctions (Rosin and Mellone 2017). It was proposed that the proteins that bind centromeric sequences, like CENP-A, also rapidly evolve, maybe to balance the effects of these phenomena, taking part in an evolutionary tug-of-war game. This was described as “centromere drive” (Zwick *et al*. 1999; Henikoff and Malik 2002).

### The centromere paradox and evolution of centromeres

From an evolutionary point of view, centromeres represent a fascinating paradigm. *Drosophila* constitutes an ideal model organism to study evolution, since many genomes have been sequenced and the phylogenetic relationships between species have been extensively determined (Kursel and Malik 2017). Although centromere function is highly conserved, centromere specification appears to be plastic as demonstrated by the lack of evolutionary conserved DNA sequences and highly divergent paralogs of CENP-A. Only the network of outer kinetochore proteins that mediate interactions with spindle microtubules reveals a higher level of evolutionary conservation. It is paradoxical that a locus with such an essential function has evolved to be present in so many different flavors. *Drosophila* has been used as a model to study not only the evolution of both dCENP-A and centromeric DNA but also the specific dCENP-A chaperone in flies, Cal1, providing important insights into the evolution of centromeres (Malik and Henikoff 2001; Phansalkar *et al*. 2012; Rosin and Mellone 2016; Kursel and Malik 2017). While, in *melanogaster*, dCENP-A is encoded by a single copy gene, two recent studies have reported that in other species, there have been five independent duplications of the genes during evolution, with the products retaining their centromeric localization but seems that their functions might be nonredundant, while some are expressed primarily in the male germline (Kursel and Malik 2017; Teixeira *et al*. 2018). Interestingly, the cenp-c gene was also found to have duplicated during evolution, something that was probably expected, given its association with Cal1 and dCENP-A (Rosin and Mellone 2016; Teixeira *et al*. 2018). Like some of the cid genes, one cenp-c duplication product also presents a male bias, raising the exciting possibility that kinetochore structure is diverse in specific contexts (Teixeira *et al*. 2018).

The gene-encoding dCENP-A (cid) was shown to have undergone adaptive evolution mostly not only in its N-terminal tail but also in part of the core region, including the L1 of the HFD (Malik and Henikoff 2001; Malik *et al*. 2002). The adaptive changes in regions that mediate DNA contacts, like L1 or the α_N_ helix on the N-terminus of the HFD, are particularly interesting and raised the exciting possibility that these changes might reflect changes in DNA binding specificity (Malik *et al*. 2002). An interesting explanation for this paradox considers the fact that centromeric DNA itself contains the sequences that are most rapidly evolving across eukaryotes (Haaf and Wilard 1997; Csink and Henikoff 1998; Murphy and Karpen 1998; Henikoff *et al*. 2001). Henikoff *et al*. (2001) proposed that these coevolution events happen in parallel to relieve centromeric imbalance, inhibit the accumulation of deleterious expansion of satellite repeats, and also provide reproductive isolation between different populations of a species leading to speciation. The latter hypothesis could, however, be rejected, since cid sequences in *simulans* and *melanogaster* strains do not correlate with the ability of these strains to rescue hybrid fitness and suggesting that evolution of the gene-encoding cid is not a cause for speciation (Sainz *et al*. 2003). Another challenge of the model suggesting that cid and centromeric DNA coevolve in response to the centromere drive was a recent study reporting that Cal1 Scm3-like region coevolves with the L1 of cid, and this concerted interplay is important for the deposition of dCENP-A into centromeric nucleosomes (Rosin and Mellone 2016). These data suggest that while centromeric sequences evolve, the L1 of cid also rapidly evolves. However, cal1 evolves at a slower rate (Phansalkar *et al*. 2012). As a result, during the intermediate stage of evolution, Cal1 cannot deposit dCENP-A to the whole length of the expanded centromeric sequences, balancing like this the possible effect of a “stronger” kinetochore due to the larger size of repeats. In the next step, Cal1 Scm3-like region also evolves to be able to deposit dCENP-A into the selected centromeric sequences (Rosin and Mellone 2016).

In addition to dCENP-A and centromeric DNA, other centromere-associated proteins in flies, like members of the condensin complex and HP1 proteins, were also shown to rapidly evolve, suggesting that they may have to compete for spindle attachment that will be optimal (Beck and Liopart 2015). Kinetochore protein evolution is greater in flies and worms, with most of the CCAN components missing from these species, and some of the outer kinetochore components are divergent compared to yeasts and mammals (Meraldi *et al*. 2006).

## Concluding Remarks

In this chapter, we tried to give an overview of our current understanding of the *Drosophila* centromere in comparison with humans and other eukaryotic organisms. Using the fruit fly as a model organism to study essential and conserved processes like chromosome segregation can reveal intriguing differences between distantly related eukaryotes. More importantly, these studies help to unmask the underlying common basic principles that connect all living beings to ensure the passing on of genetic material from one generation to the next.
